# A bioprocessing approach for osteogenic differentiation: combining electrical stimulation with the F1 protein fraction from natural latex of *Hevea brasiliensis*

**DOI:** 10.1007/s00449-026-03366-y

**Published:** 2026-06-25

**Authors:** Rafaela Canassa Maiolini, Ana Beatriz do Amaral Oliveira, João Lucas de Oliveira Dantas, Fábio Augusto Bispo Júnior, Guilherme Ferreira Caetano, Ricardo José de Mendonça, Patrícia Soares Santiago

**Affiliations:** 1 Institute of Chemistry, Paulista State University “Júlio de Mesquita Filho” (UNESP), Araraquara, SP Brazil; 2 University Center of Hermínio Ometto Fundation (FHO), Araras, SP Brazil; 3 Department of Clinical Medicine, School of Medicine at Ribeirão Preto, University of São Paulo, Ribeirão Preto, SP Brazil; 4 Department of Biochemistry, Pharmacology and Physiology, Federal University of Triângulo Mineiro (UFTM), Uberaba, Minas Gerais Brazil; 5 Faculty of Agricultural Sciences of Vale da Ribeira, Paulista State University “Júlio de Mesquita Filho” (UNESP), Registro, SP Brazil; 6 Institute for Advance Studies of the Sea (IEAMAR), Paulista State University “Júlio de Mesquita Filho” (UNESP), São Vicente, SP Brazil; 7 Graduate Program of Orthodontics, University Center of Hermínio Ometto Foundation (FHO), Araras, SP, Brazil

**Keywords:** Electrical stimulation, Natural latex, Tissue engineering, Stem cells, Bone repair

## Abstract

The rising life expectancy and aging of bones have created a demand for new treatments for fractures and bone loss. Tissue engineering and development of scalable bioprocesses are emerging as a promising solution for tissue repair. This study aimed to evaluate in vitro proliferation and osteogenic differentiation of mesenchymal stem cells (MSCs) from the bone marrow of Wistar rats subjected to electrical stimulation (ES) and cultured with F1 protein fraction from the latex of the *Hevea brasiliensis* rubber tree. Additionally, the study aimed to analyze the expression of genes related to osteogenesis. Cell viability (MTT) and osteogenic differentiation (Alizarin red) assays were performed using ES of 60 s, 150 s, and 300 s at 10 μA. The medium was supplemented with F1 at concentrations of 1%, 0.1%, and 0.01%. For gene expression analyses, cells were stimulated for 60 s and 300 s and supplemented with 0.001% and 0.0001% F1. All experimental conditions demonstrated cell viability above 70% with no cytotoxicity. Osteogenic differentiation exceeded 80%, with the ES-60 s group combined with 0.0001% F1 showing the highest efficacy. Expression of *Bmp2* and *Ltype* genes increased with 60 s F1-0.001% and 0.0001%. *Col1a1* gene expression was higher with F1-0.001% and ES-300 s, while *Alp* and *Runx2* were prominent with F1-0.0001%. *Camk2* showed increased expression with F1-0.001% at 60 s and 300 s. F1 protein fraction of latex has a significant biological effect on promoting the proliferation and osteogenic differentiation of MSCs, especially when combined with ES. This highlights its potential as a therapeutic option in regenerative medicine for bone repair.

## Introduction

**Fig. 1:**
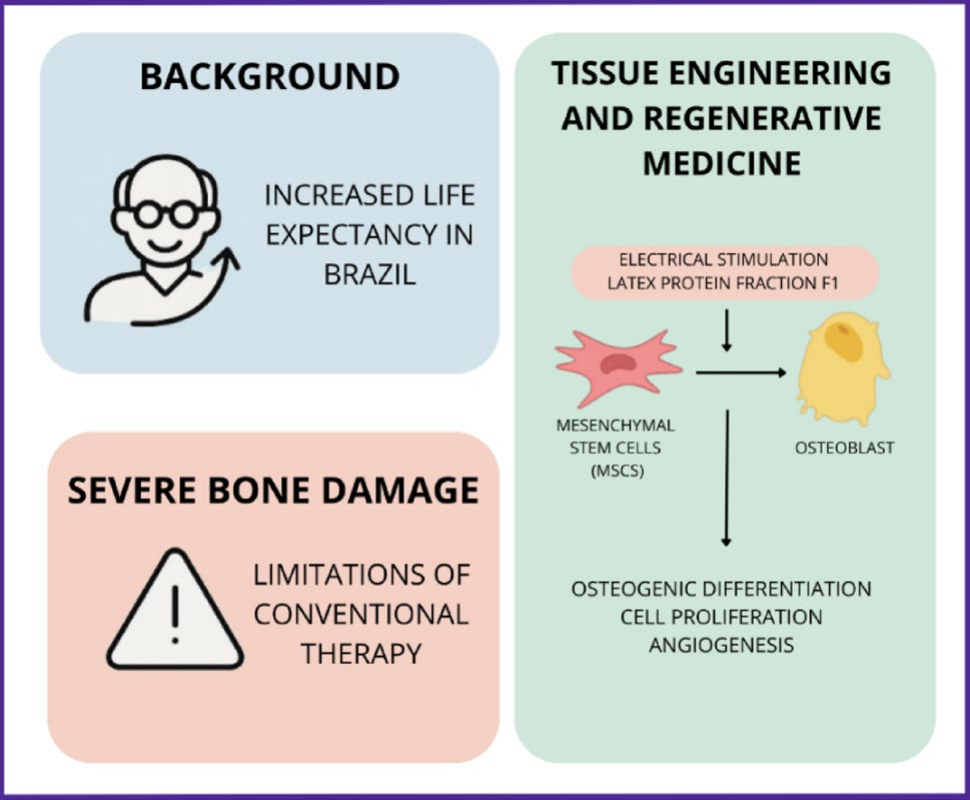
Introduction Conceptual Outline. Source: Authors

The rise in life expectancy in Brazil, as projected by the Brazilian Institute of Geography and Statistics [1], has increased the importance of research on aging and bone tissue repair. Currently, conventional treatments are based on transplants, such as autografts, allografts, and xenografts. However, these approaches have significant clinical limitations because they are invasive and limited by a lack of tissue supply or the need for additional surgeries. These factors increase patient discomfort and recovery time, as well as raise the risks of infection and rejection, negatively impacting morbidity and mortality rates [2, 3]. Thus, tissue engineering and regenerative medicine have emerged as promising alternatives, using stem cells, biomaterials and biological and biophysical stimuli to repair and regenerate damaged tissues (Fig. 1).

Mesenchymal stem cells (MSCs) have the ability to self-renew, multipotent differentiation and are commonly sourced from bone marrow. In the treatment of bone injuries, the in vitro differentiation of patient MSCs using three-dimensional supports and inducing factors such as cytokines and electrical stimulation (ES) has become increasingly important [4, 5].

The use of electrical stimulation (ES) in MSC culture shows promising possibility for bone tissue repair. ES takes advantage of bone piezoelectricity to promote MSC differentiation into osteoblasts, cell proliferation, growth factor synthesis, and angiogenesis [6]. Electrical stimulation enhances bone repair in animals by promoting the migration and osteogenic differentiation of mesenchymal stem cells (MSCs) through modulation of calcium and other ion pathways. The specific mechanisms of electrical stimulation (ES) are not fully understood, but it is believed to regulate Ca2 + , Na + , K + and Cl- pathways [6].

Therapy can enhance tissue repair, reduce inflammation, and promote vascularization and collagen organization [7, 8]. Studies indicate that currents ranging from 10 µA to 50 µA are beneficial for osteoblast therapy and the production of growth factors for bone formation in the tibia and femur of dogs and rabbits. Currents exceeding 50 µA may lead to bone necrosis [9].

F1 protein fraction (LXHb) from the rubber tree *Hevea brasiliensis* is a valuable resource in tissue engineering promoting healing due to its angiogenic properties, it acts as a growth factor, it is cost-effective and easy to isolate an work with. Guerra et al. demonstrated that applying latex membranes to injured tissues released a VEGF-like substance, promoting the regeneration of blood vessels for nutrient transport [10].

Therefore, the present study aimed to explore the combined effects of electrical stimulation and the F1 protein fraction of natural latex on bone marrow MSCs to promote their proliferation and osteogenic differentiation.

## Materials and methods

**Fig. 2:**
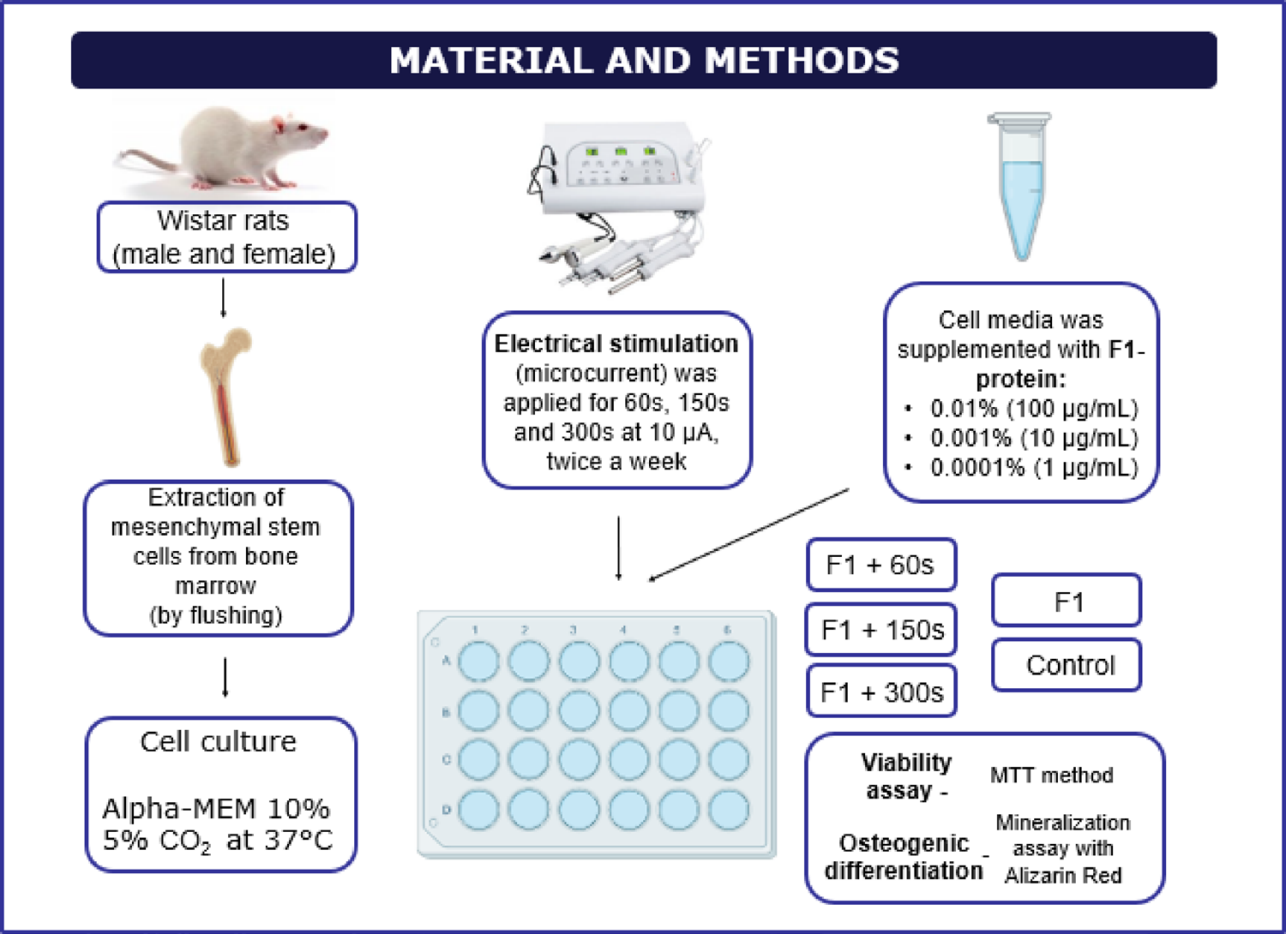
Experimental design of the study. Source: Authors

All cell culture procedures involving stem cell extraction were approved by the Ethics Committee (No.014/2022) for the Use of Animals of the Hermínio Ometto Foundation University Center FHO/Araras. The experimental scheme is illustrated in Fig. 2.

### Isolation and culture of mesenchymal stem cells (MSCs)

Bone marrow-derived mesenchymal stem cells (MSCs) were isolated from the femurs of 60-day-old Wistar rats. The rats were euthanized using anesthesia with 10% ketamine hydrochloride (30 mg.kg-1 body weight) and 2% xylazine hydrochloride (10 mg.kg -1 body weight), followed by cervical dislocation. The femurs were cleaned by removing muscle and connective tissue, then placed in a conical tube with 15 mL of PBS (Invitrogen, Waltham, MA, USA). Afterward, they were transferred to a new tube with 70% ethanol for 5 min. The femoral epiphyses were then cut using sterile forceps and scissors. Using the flushing technique, the 21G syringe needle was inserted into the diaphysis and the entire bone marrow was washed with 10% Alpha MEM culture medium (1% glutamine, 1% penicillin/streptomycin, supplemented with 10% FBS) (Gibco-Invitrogen). The solution was centrifuged, and the pellet was resuspended in 1 mL of the same culture medium. The cells were then cultured in 75 cm2 culture bottles in an incubator with 5% CO2 and 95% air at 37 °C. The medium was changed twice a week, and passages were performed when the cells reached 80% confluence. Cells from the 4th, 5th, or 6th passages were used in the experiment.

### Morphological characterization of mesenchymal stem cells (MSCs)

To asses multipotency, bone marrow MSCs were cultured in vitro and induced to differentiate into adipocytes and osteoblasts for 14 days. The cells were divided into three groups (cultured in osteogenic medium, adipogenic medium and basal medium) in 24-well culture plates at a concentration of 1 × 104 cells/well. Adipogenic differentiation was induced by adding adipogenesis-inducing medium (α-MEM medium with 10% FBS, 1 μM dexamethasone, 10 μg/mL insulin, 100 μM indomethacin, 1% glutamine, and 1% penicillin/streptomycin) to the designated wells. For osteogenic differentiation, cells were cultured in osteogenesis-inducing medium (α-MEM supplemented with 7.5% FBS, 0.1 μM dexamethasone, 200 μM ascorbic acid, 10 mM β-glycerol phosphate, 1% glutamine, and 1% penicillin/streptomycin) as described by CAETANO et al., 2018. Control wells were cultured in basal medium (α-MEM medium supplemented with 7.5% FBS, 1% glutamine, 1% penicillin/streptomycin). The plates were incubated under standard conditions (37 °C under 5% CO 2 and 95% humidity), and the media were changed three times a week. After 14 days, all culture wells were washed with PBS. Adipogenic differentiation wells were stained with 2% Sudan IV, and osteogenic differentiation wells were stained with 0.2% Alizarin Red (ARS).

### Application of electrical stimulation and latex F1 protein fraction

Approximately 1 × 10^4^ MSCs were individually suspended in 500 µL of supplemented α-MEM culture medium (10% FBS, 1% L-glutamine, and 1% antibiotic-antifungal) and seeded in 24-well culture plates. After 24 h of cell adhesion, the culture was replaced with 1 mL of 7.5% α-MEM medium (supplemented 7.5% FBS, 1% L-glutamine, and 1% antibiotic-antifungal) or osteogenic medium (the same mentioned above), according to the proposed assay.

The lyophilized F1 protein fraction was accurately weighed and resuspended in PBS buffer (pH 7.4) to obtain stock solutions at concentrations of 10 mg/mL, 1 mg/mL, and 0.1 mg/mL, which were sterilized by filtration through a 0.22 µm membrane to ensure the absence of aggregates. The composition of the F1 fraction was characterized by elemental analysis (CNS 928 Analyzer, LECO), identifying a nitrogen content of 1.633%, corresponding to 10.2% total protein. It should be noted that, in this study, the concentrations described in the in vitro experiments refer to the total mass of the fraction F1 and not only to its protein content. Subsequently, 10 µL of each F1 stock solution were added to the culture wells (final volume of 1 mL) to achieve final concentrations of 0.01% (100 µg/mL), 0.001% (10 µg/mL), and 0.0001% (1 µg/mL), respectively.

Electrical stimulation (ES) was applied directly and continuously using a low-intensity transcutaneous device (Physiotonus Microcurrent, BIOSET®, Indústria de Tecnologia Eletrônica Ltda., Rio Claro, SP, Brazil). Two electrodes connected to the device were sterilized by immersing them in 70% alcohol for 10 min, drying them with sterile gauze, and then carefully submerged in the culture medium. The cells were exposed to different durations of ES application (60 s, 150 s, and 300 s) twice a week at an intensity of 10 µA. The culture medium was changed every 3 days.

### Cell viability analysis—MTT method

Cell viability analysis of MSCs was assessed using the colorimetric MTT assay (Sigma-Aldrich, Dorset, UK). Metabolically active cells are capable of reducing the chemical compound 3-(4,5-dimethylthiazol-2-yl)-2,5-diphenyltetrazolium bromide into purple formazan crystals, which are soluble in DMSO. MSCs were cultured in 24-well plates with 7.5% α-MEM basal culture medium supplemented with 7.5% FBS, 1% L-glutamine, and 1% antibiotic-antifungal. Five groups were prepared for each of the three concentrations of the F1 fraction: i. F1 associated with ES (60 s); ii. F1 associated with ES (150 s); iii. F1 associated with ES (300 s); iv. F1 only; v. control (no F1 or ES). After 7 days, the wells were washed with PBS and treated with 300 µL of MTT stock solution (5 mg.mL-1 in PBS). The plates were incubated for 3 h in the dark. After removing the MTT solution, 300 µL of dimethyl sulfoxide (DMSO) was added. The optical density was measured at a wavelength of 540 nm using a Biotek ELISA reader. The absorbance value was directly proportional to the number of viable cells. Cell metabolic activity was normalized to the control group (without ES and F1), which was considered as 100% relative viability.

### Osteogenic differentiation of MSCs

MSCs were cultured in 1 mL of osteogenesis-inducing medium (as previously described). The experimental groups were the same as those used in the viability assay. After 14 days, osteogenic differentiation was evaluated by staining with Alizarin Red (0.2% ARS) for 15 min. After the staining and washing steps, the bound dye was eluted by adding 300 µL of 10% (v/v) acetic acid to each well. The optical density was measured at a wavelength of 450 nm using a Biotek ELISA reader. The amount of mineralization was directly proportional to the absorbance value. The results were calculated based on cells from the control group (without ES and F1) as 100% relative mineralization. Osteogenic differentiation assays were performed using only electrical stimulation (60 s, 150 s, 300 s) and using only the F1 protein fraction (0.01%, 0.001%, 0.0001%) individually.

### Gene expression of mesenchymal stem cells (MSCs)

Genes associated with osteogenesis (*Runx2*; *Bmp2*; *Alp*; *Col1a1*; *Camk2*; *Ltype*) were analyzed using RT-PCR. Cultured MSC samples were collected by trypsinization with 0.05% trypsin solution (Sigma-Aldrich), and RNA isolation was carried out with TRIzolTM reagent (Invitrogen, Waltham, MA, USA). RNA concentration and quality were assessed using a spectrophotometer based on A260/280 and A260/230 ratios. cDNA was synthesized from 100 ng of total RNA using the High Capacity kit (Invitrogen, Waltham, MA, USA). Normalization was done with the endogenous gene Eif2b1, and the results were calculated using the 2^−ΔΔCT^ method.

### Statistical analysis

Cell viability and osteogenic differentiation assays were conducted in triplicate and repeated three times. Statistical analysis was performed using one-way ANOVA (One-way ANOVA with Tukey’s post-test and Kruskal–Wallis with Dunn’s post-test). Data were presented as mean ± standard error of the mean using Graphpad Prism 8.0 software (USA) for testing and graph construction. Significance was considered at p < 0.05 (less than 5%).

## Results

### Morphological characterization of mesenchymal stem cells (MSCs)

**Fig. 3:**
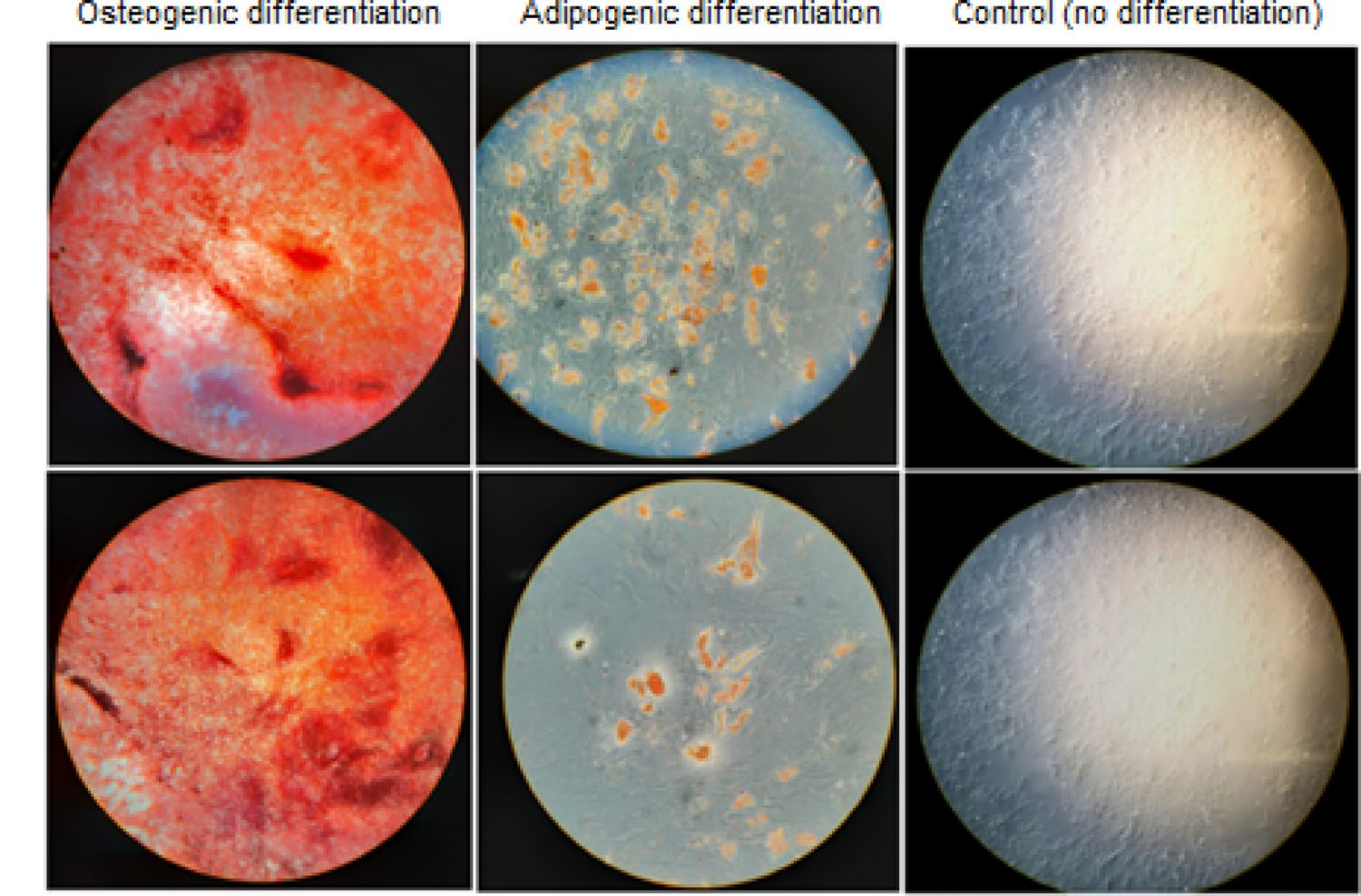
In vitro differentiation of bone marrow-derived mesenchymal stromal cells into osteoblasts (osteogenic differentiation) and adipocytes (adipogenic differentiation) compared to the control group (no differentiation) observed under a microscope at 100 × magnification. Source: Authors

Figure 3 demonstrates the in vitro differentiation potential of bone marrow-derived MSCs. After 14 days of adipogenic and osteogenic induction, the cells differentiated into adipocytes and osteoblasts, respectively. A control group was cultured in 7.5% α-MEM basal medium. The images were captured using inverted microscopy.

### Viability of bone marrow-derived MSCs

**Fig. 4:**
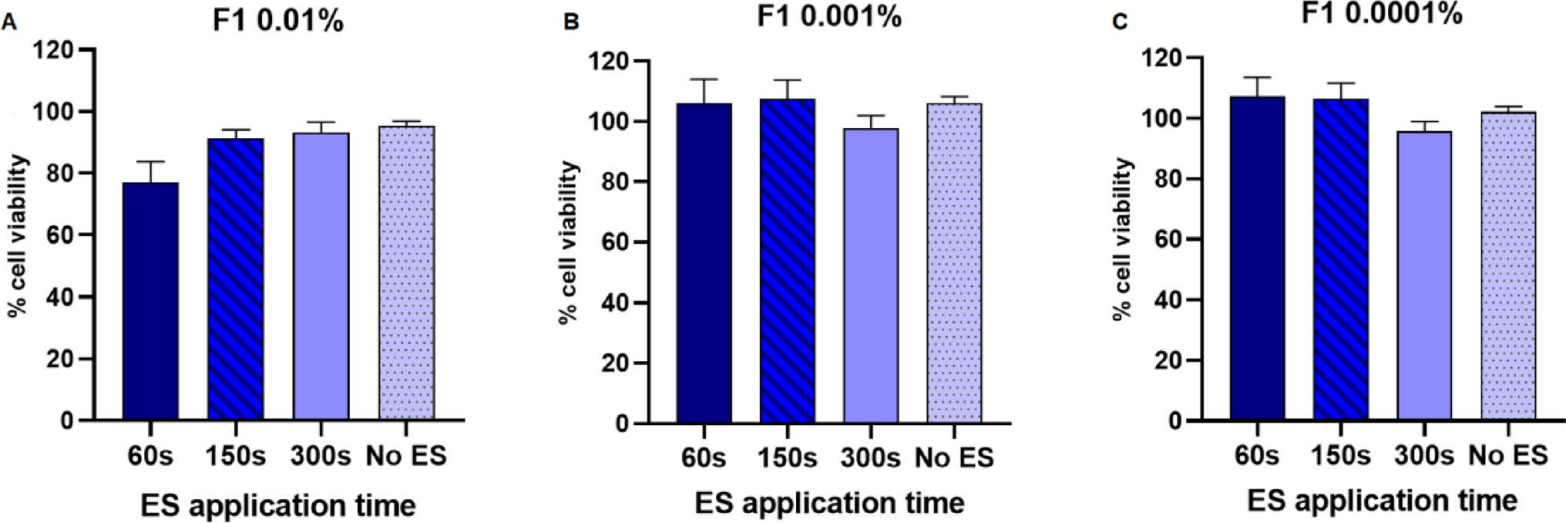
Evaluation of cell viability (MTT) of MSCs after culture with F1 protein fraction at different concentrations and electrical stimulation twice a week at application times of 60 s, 150 s, and 300 s at 10 μA after 7 days. ** A ** F1 application at 0.01%. ** B ** F1 application at 0.001%. ** C ** F1 application at 0.0001%. Data are expressed as a percentage relative to the control group (100%). Statistical significance (p < 0.05) analyzed using One-way ANOVA, Tukey post-test, and Kruskal–Wallis test with Dunn post-test. Source: Authors

MSC cell viability was evaluated using the MTT method with the F1 fraction and electrical stimulation alone or in combination. After 7 days of treatment, the highest concentration of F1 0.01% (Fig. 4A), resulted in decreased cell viability in all experimental groups compared to the control (100%). At this concentration, the group treated with only the protein fraction had the highest viability (95%). Combining F1 and ES for 60 s (the shortest application time) showed the lowest cell viability (77%) compared to other ES durations. At F1 concentrations of 0.001% and 0.0001%, similar results were observed (Fig. 4B and C). The fraction alone and in combination with ES for 60 s and 150 s showed viability rates above 100% at both concentrations. However, the combination of these two concentrations increased the viability rate compared to the unstimulated group. The group with an ES duration of 300 s showed the lowest rates (0.001%—97.8% and 0.0001%—95.7%), which were lower than the control group. However, there were no significant differences between the experimental groups. All tested groups had cell viability above 70%, as required for in vitro tests.

### Osteogenic differentiation of bone marrow-derived MSCs

**Fig. 5:**
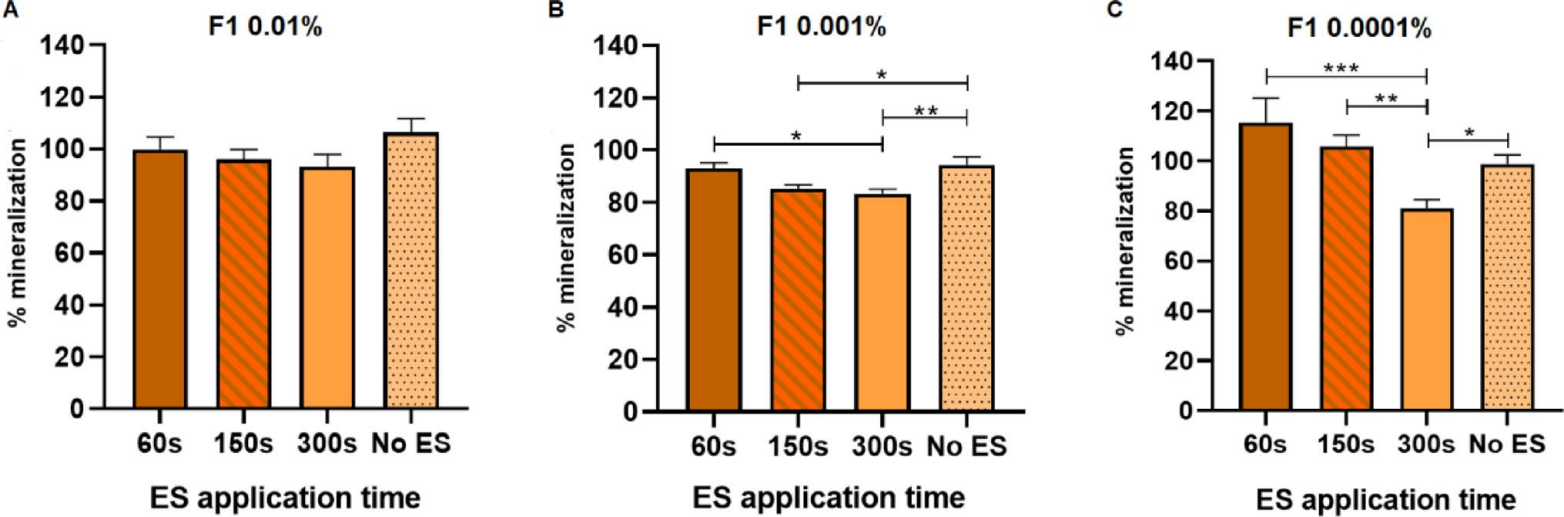
In vitro mineralization assay of bone marrow-derived MSCs cultured in osteogenic induction medium with electrical stimulation and application of P1 protein fraction. ** A ** 0.01% F1 application. ** B ** 0.001% F1 application. ** C ** 0.0001% F1 application. Data are expressed as a percentage relative to the control group (100%). Statistical significance (p < 0.05) analyzed using One-way ANOVA, Tukey post-test, and Kruskal–Wallis test with Dunn post-test. Source: Authors

**Fig. 6:**
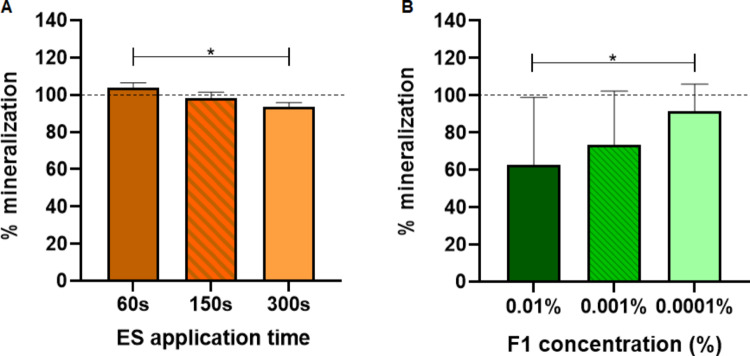
In vitro bone marrow-derived MSC mineralization assay in osteogenic medium. ** A ** Application of electrical stimulation only. ** B ** Application of F1 protein only. Data are expressed as a percentage relative to the control group (100%). Statistical significance (p < 0.05) analyzed with One-way ANOVA, Tukey post-test and Kruskal–Wallis test with Dunn post-test. Source: Authors

MSCs were cultured for 14 days in osteogenic medium with different concentrations of the F1 protein fraction of natural latex (0.01%, 0.001%, and 0.0001%) and stimulated with microcurrent (60 s, 150 s, and 300 s). Regardless of the F1 concentration, the results showed that longer stimulation durations led to reduced mineralization, indicating decreased osteogenic differentiation. The groups supplemented with F1 at 0.01% (Fig. 5A) did not show a statistically significant difference, and the highest mineralization rate was observed in the unstimulated group (106%). When F1 was used at 0.001% (Fig. 5B), none of the experimental groups showed rates higher than the control. However, applying ES for 60 s resulted in a 10% increase in mineralization compared to ES for 300 s. The groups supplemented with F1 at 0.0001% (Fig. 5C) showed rates higher than 100% in association with ES for 60 s and 150 s, with better results in the shortest application time (116%). The differentiation tests performed only with the application of microcurrent or the protein fraction confirmed these results, showing that reducing the ES duration from 300 to 60 s (Fig. 6A) and decreasing the F1 concentration from 0.01% to 0.0001% (Fig. 6B) increased the mineralization rate.

### Gene expression

**Fig. 7:**
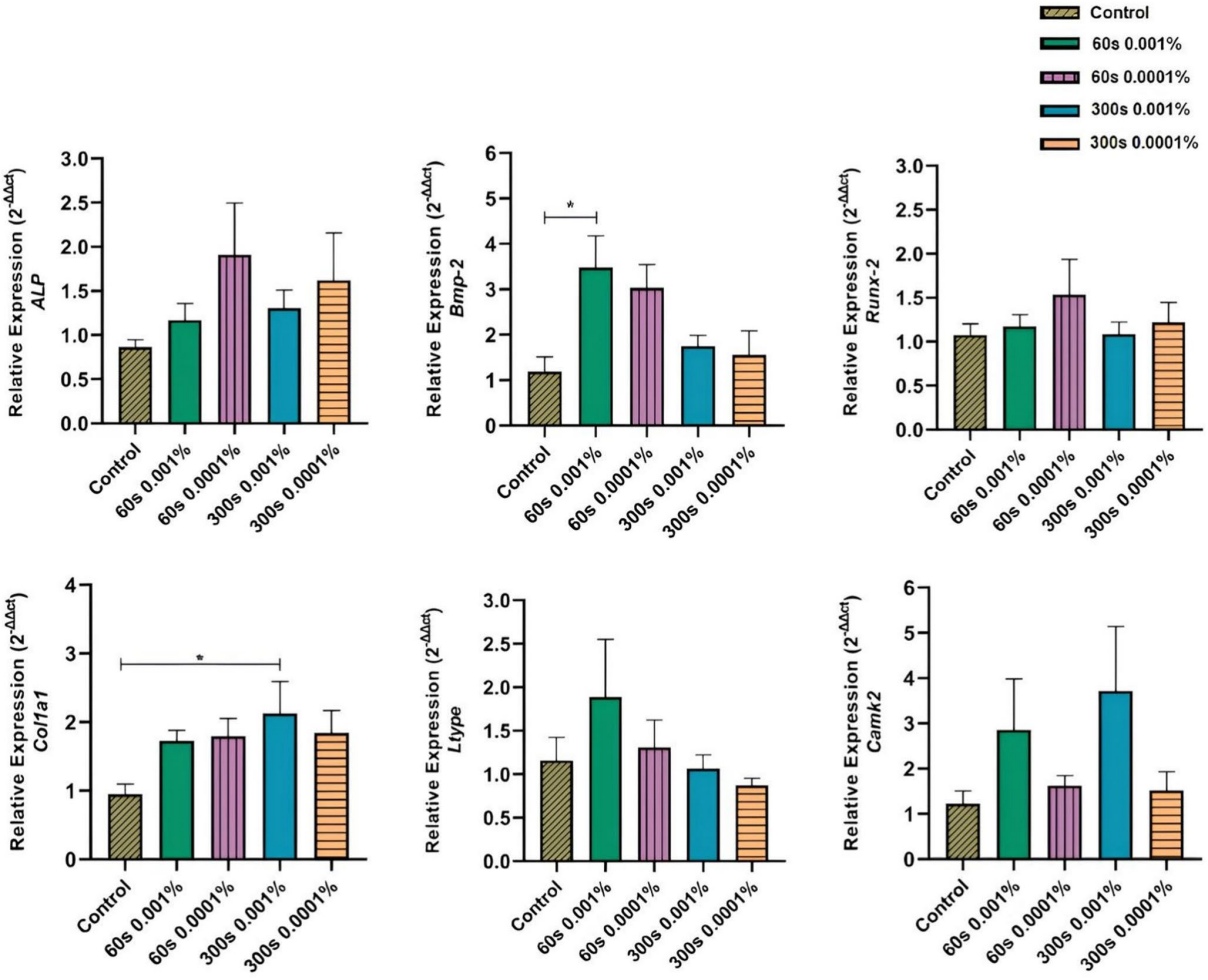
Relative expression of ** A** *ALP*, ** B** *BMP2*, ** C** *RUNX2*, ** D** *COL1A1*, ** E** *LTYPE*, ** F** *CAMK2* (2 ^−ΔΔct^). * Statistical significance (p < 0.05) was determined using One-way ANOVA, Tukey post-test and Kruskal–Wallis test with Dunn post-test. Source: Authors

Based on the optimal outcomes from the methods outlined above, electrical stimulation durations of 60 s and 300 s, along with F1 fraction concentrations of 0.001% and 0.0001%, were selected to assess the gene expression related to osteogenic differentiation (*Alp*, *Ltype*, *Runx2*, *Col1a1*, *Bmp2*, *Camk2*). The results are presented in Fig. 7 and Table 1, indicating that the *Alp* and *Ltype* genes exhibited higher expression levels in the 60 s 0.001% group. The *Col1a1* gene exhibited higher expression levels in the 300 s 0.001% group. *Runx2* and *Bmp2* showed increased expression in the 60 s stimulation groups for both F1 concentrations. In contrast, the *Camk2* gene showed increased expression in the groups treated with F1 at 0.001% for 300 s.

**Table 1:** Gene expression related to osteogenic differentiation in MSC cultures stimulated for 60 s and 300 s supplemented with the F1 fraction at 0.001% and 0.0001%. Source: Authors

Gene	Function	Main Results
*ALP*	Early marker of osteogenic differentiation, aiding in the maturation of pre-osteoblasts into differentiated osteoblasts [11]	Group 60 s 0.0001% showed higher expression, followed by group 300 s 0.001%
*RUNX2*	Regulator of bone formation, mediator of activation and/or temporal repression of cell growth, present in the formation of immature bone [12]	Increasing ES time led to a decrease in expression in both F1 concentrations
*BMP2*	The bone morphogenetic protein BMP-2 is a marker that stimulates skeletal system growth, development, and bone regeneration [13]. It activates intracellular signaling molecule Runx2 [14]	In both F1 concentrations, longer ES time led to decreased expression. The 60 s 0.001% group showed a significant increase compared to the control group
*COL1A1*	Expression of type 1 collagen (80% of bone protein content) [15]	Group 300 s 0.001% showed higher expression, with a significant difference in relation to the control. Groups 60 s had lower and similar expression among them
*LTYPE*	Expression of the calcium channel (voltage-dependent), plays a crucial role in regulating calcium ion influx into bone cells [16]	The expression was higher in the 60 s group at 0.001% and lower in the 300 s group at 0.0001%. The increase in ES and the lower concentration of F1 resulted in reduced expression
*CAMK2*	Expression of the cytoplasmic protein calmodulin (CaM). Ca2 + /CaM binding promotes calcineurin signaling, increasing osteogenesis gene signaling [8]	The highest F1 expression was found in the 300 s group at 0.001%, followed by the 60 s group at 0.001%. Groups with lower F1 concentrations exhibited reduced values

Statistical significance (p < 0.05) determined using One-way ANOVA, Tukey post-test, and Kruskal–Wallis test with Dunn post-test

## Discussion

Several studies have examined how MSCs respond to ES, nevertheless the mechanisms of interaction between stimulation and cells remain unclear, highlighting the need for further research and standardization of application methods [17]. Research on the natural latex fraction has shown that F1 plays a crucial role in osteogenic differentiation by promoting neoangiogenic activity and increasing vascular permeability [18, 19]. The addition of F1 at 0.01% in graphene and polycaprolactone scaffolds enhanced stem cell proliferation and osteogenic differentiation [20].

Studies have demonstrated that biomembranes derived from *Hevea brasiliensis* latex promote the healing of acute dermal ulcers by facilitating early epithelialization, collagen fiber organization, and new vessels [21]. MSCs are undifferentiated and unspecialized cells that are easily isolated [22]. Several studies have shown promising results in inducing osteogenic differentiation in bone marrow MSCs through stimulation or the F1 fraction [20, 23]. Therefore, this study aims to assess the viability and osteogenic differentiation potential of bone marrow-derived MSCs in combination with ES and the F1 protein fraction.

The identity of MSCs was confirmed according to the functional criteria established by the International Society for Cellular Therapy (ISCT) [24], based on their potential to differentiate into adipogenic and osteogenic lineages. Although immunophenotyping by flow cytometry was not performed, future studies will include surface marker profiling to further support phenotypic characterization. In our results, we assessed the viability of MSCs using the MTT assay following ISO 10993 (International Standard Organization) guidelines.

MSCs were stimulated for different durations (60, 150, and 300 s) and cultured in medium containing F1 (0.01%, 0.001%, and 0.0001%), results showed no cytotoxicity in any experimental group, with over 70% of cells remaining viable. An increase in the viability rate was observed as the F1 concentration and the ES application time were reduced. Previous studies also reported that the latex protein fraction did not present cytotoxicity and demonstrated significant angiogenic and tissue repair properties, favoring cell proliferation, especially at concentrations of 0.1 mg.mL-1 (0.01%) and 0.01 mg.mL-1 (0.001%) [20, 25, 26]. Furthermore, in vitro application of ES between 10 µA and 50 µA did not induce cell death nor affect cell proliferation [27].

Therapy with F1 and ES affects the osteogenic differentiation of MSCs. Mineralization assays that this association showed a higher rate in the 0.0001% at 60 s group. However, when stimulation was applied for 300 s, the results were opposite, possibly suggesting that the mesenchymal cells may decrease their differentiation potential with longer stimulation supplemented with higher F1 concentration. Studies comparing the effects of different concentrations of the protein fraction found that F1 at 0.0001% w/v accelerated tissue regeneration and wound closure, promoting the healing process [28].

This study sought to deepen the molecular mechanisms of osteogenic differentiation by analyzing the expression of specific osteogenic markers. The Wnt/β-catenin signaling pathway plays a crucial role in regulating osteoblast differentiation during the reparative phase of osteogenesis process. It consists of the canonical Wnt signaling pathway, which relies on β-catenin (Wnt/β-catenin pathway) function and promotes repair, and non-canonical Wnt pathways that have varying effects on osteogenesis based on stem cell maturation stage [29].

Activation of the Wnt/β-catenin pathway can promote the expression of the *Runx2* gene [12]. Studies on the in vitro osteogenic response of MSCs found no significant difference in *Runx2* gene expression between groups after 7 and 14 days of the experiment, as well as in the results of this study. This suggests that gene expression decreases in mature osteoblasts [30]. The *Bmp2* gene encodes the BMP-2 protein, which promotes osteoblast differentiation and initial bone formation by activating signaling molecules and markers [31]. Studies on rat calvarial fractures treated with latex reported a decrease in Bmp2 expression over time, mainly attributed to the production of sclerostin by osteocytes, a potent antagonist protein that competes for BMP-2 binding sites [32]. Though direct measurement of sclerostin expression was not performed in this study, the results observed in the ES 300 s groups suggest the occurrence of a similar inhibitory mechanism, in which prolonged electrical stimulation may have induced excessive expression of sclerostin in the bone cell membrane, resulting in decreased *Bmp2* expression.

*ALP* is considered an early marker. Previous studies have observed significant increased expression of this gene in stimulated MSCs, mainly on the 7th and 14th day of testing, consistent with the results of this study. Voltage-dependent calcium channels, regulated by the *L-type* gene, when stimulated, play a crucial role in bone strength by facilitating calcium deposition for hydroxyapatite complex formation [16]. Stimulation is believed to depolarize and alter the conformation of these channels, which would imply a shift in calcium influx [9]. The binding of this ion promotes a conformational change in the calmodulin enzyme, triggering phosphorylation processes essential for biological functions in cell differentiation [33]. The literature indicates that stimulation enhances the expression of calcium signaling genes and promotes mineralization after 14 days.

However, our study did not find a consistent correlation between the results of *L-type* gene expression and *Camk2*, as the responses varied based on the duration of microcurrent exposure or the concentration of the fraction used. It is suggested that the intracellular calcium concentration influences the subcellular distribution of calmodulin, as well as its association with target proteins, and the activation of specific targets in its conformational state [34].

## Conclusion

The morphological characterization of mesenchymal stem cells from Wistar rat bone marrow showed successful in vitro isolation, promoting cell proliferation and differentiation. Treatment with the F1 fraction at concentrations of 0.01%, 0.001% and 0.0001% and electrical stimulation at 10 µA for 60 s, 150 s and 300 s did not show in vitro cytotoxic and enhanced osteogenic differentiation of the cells. The gene expression analysis showed that combining electrical stimulation with the F1 protein fraction enhanced the expression of bone markers involved in the early stages of osteogenesis, particularly in the 60 s-0.0001% group. Genes associated with mineralization, especially the Ca2 + /CaM pathway, were more active in the groups treated with F1 at 0.001%. Stem cells treated with a 10 µA microcurrent for 60 s and supplemented with 0.0001% F1 showed the highest mineralization rate compared to other groups, including the control group (without F1 and without ES). These findings suggest a promising potential for using this combination as a future treatment and biotechnological approach in bone repair and tissue engineering. Thus, it may support the development of scalable bioprocesses for MSC differentiation under defined physical and biochemical conditions.

## Data Availability

The datasets generated and/or analyzed during this study are available upon request from the corresponding author.
